# The Bonding State and Surface Roughness of Carbon-Doped TiZrN Coatings for Hydrogen Permeation Barriers

**DOI:** 10.3390/nano13212905

**Published:** 2023-11-05

**Authors:** Seonghoon Kim, Taewoo Kim, Seungjae Lee, Heesoo Lee

**Affiliations:** 1 School of Materials Science and Engineering, Pusan National University, Busan 46241, Republic of Korea; seonghoonkim@pusan.ac.kr (S.K.); bluejae2@pusan.ac.kr (S.L.); 2 Nuclear Power Industry Division, Korea Institute of Energy Technology Evaluation and Planning, Seoul 02792, Republic of Korea; twkim@ketep.re.kr

**Keywords:** laser carburization, intergranular structure, amorphization, amorphous carbon-embedded nanocomposite, surface topography, hydrogen permeability

## Abstract

We doped carbon into a TiZrN coating to reduce hydrogen permeability, and investigated the phase formation, bonding state, microstructure, and surface roughness of the carbon-doped TiZrN. The laser output for laser carburization was limited to a range of 20–50%. The grain size of the TiZrN coatings decreased from 26.49 nm before carburization to 18.31 nm after carburization. For XPS analysis, the sp^2^/sp^3^ ratio was 1.23 at 20% laser output, but it showed 2.64 at 40% laser output, which means that amorphous carbon was formed. As the grain size decreased with the formation of amorphous carbon, the surface microstructure of the carbon-doped TiZrN coatings transitioned to an intergranular structure, indicating the creation of amorphous carbon-embedded (Ti, Zr)(C, N) in the coating. The surface roughness (Ra) of the carbon-doped TiZrN coating was decreased to a maximum of 7.12 nm, and the hydrogen permeability correspondingly decreased by 78% at 573 K.

## 1. Introduction

Hydrogen is an eco-friendly energy vector because it does not emit greenhouse gases in the process of converting into electrical energy. To realize a hydrogen society, various technical problems that are caused by the material characteristics of hydrogen must be solved [1,2,3]. It is well known that the presence of hydrogen within metal materials such as carbon steel, stainless steel, and aluminum alloys can degrade the mechanical properties of the material. When steel is exposed to hydrogen gas, hydrogen molecules are relatively easily dissociated at the surface of the steel and penetrate into the grain, causing the material to be attacked and inducing hydrogen embrittlement [4,5,6]. Hydrogen embrittlement (HE) refers to mechanical damage to a metal due to the penetration of hydrogen into the metal, causing a loss in ductility and tensile strength. HE can occur due to the corrosion of steel by H_2_S when hydrogen atoms are generated. During the corrosion of steel in geothermal steam, H_2_S reacts with the surface and forms a corrosion film (FeS, MnS) and free hydrogen ions (H^+^). The free hydrogen ion would normally not diffuse into the metal, but the sulfide (S2^−^) ion acts as a poison and promotes the uptake of the hydrogen, which becomes trapped in the metal structure and results in the embrittlement of the metal. [7,8].

In the late 1970s, Fowler et al. proposed the concept of a hydrogen permeation barrier by covering materials with one or more layers of a coating with a low hydrogen permeability to prevent or delay the permeation and diffusion of hydrogen without changing the desirable attributes of the base materials [9,10,11]. Studies on hydrogen permeation barrier coatings have been conducted in various fields, such as nuclear fusion reactors, fuel cells, corrosion-resistant components, and vacuum equipment, and fine ceramic coatings, such as Al_2_O_3_, TiC, TiN, and BN, have been reported to be suitable as hydrogen permeation barriers [12,13,14]. Zhang et al. prepared an aluminum-rich coating on HR-2 steel that can reduce deuterium permeation by two to three orders of magnitude. He et al. deposited an Al_2_O_3_/Cr_2_O_3_ composite film on 316 L stainless steel via metal–organic chemical vapor deposition (MOCVD). Their composite films can significantly reduce deuterium permeation [15,16].

To enhance the performance of tools, researchers have studied coatings made of transition metal nitrides and carbides, known for their excellent wear resistance and hardness. However, binary coatings like TiN and TiC are not always suitable for harsh operating conditions. As a result, various industries are turning to ternary or multiphase coatings [17,18]. The field of multiphase and nanocomposite coatings is actively investigated, as these coatings exhibit exceptional properties due to their unique structural interactions and diverse microstructures [19]. The incorporation of light elements, such as carbon and nitrogen, plays a vital role in solid solution strengthening. Carbon is a widely adopted element for enhancing the surface characteristics of coatings. Researchers have extensively studied the impact of carbon compositions on surface properties, with Jinlong Li et al. adjusting the C_2_H_2_ flow rate to create gradient Ti(C,N) films and observing changes in wear resistance. Similarly, J. M. Lackner et al. deposited and characterized (Ti, Al)(C, N) coatings with varying carbon contents, noting that a higher carbon content results in carbon precipitation and reduced friction [20,21].

In the case of carbon doping via laser carburization, the thermal energy from the laser serves as a source of energy for carbon diffusion. The behavior of carbon, both interstitial and substitutional, undergoes changes as the thermal energy of the laser is increased. This leads to the accumulation of amorphous carbon once the limit of carbon solid solution is reached [22,23]. Amorphous carbon, with its excellent properties such as high hardness, high wear resistance, a low friction coefficient, chemical inertness, high gas barrier properties, and biocompatibility, is widely used as a surface protective coating in addition to ceramic coatings [24,25,26,27]. Amorphous carbon coatings are known to exhibit varying properties depending on the ratio of sp^2^, sp^3^, and hydrogen within the coating. In particular, the proportion of sp^2^/sp^3^ hybrid bonds can significantly influence these properties. For instance, as the ratio of sp^2^ hybrid bonds increases within the coating, there is an observed increase in clustering phenomena. These clusters consist of sp^2^ hybrid bonds with delocalized electrons, leading to enhancements in the coating’s electrical and optical properties. However, a higher sp^2^ ratio may result in a decrease in hardness. Conversely, when the proportion of sp^3^ hybrid bonds is increased, mechanical properties are often enhanced. Yet, this can also lead to a rise in the band gap, resulting in a decrease in electrical properties. Hence, the sp^2^/sp^3^ ratio exerts a substantial influence on the physicochemical properties of a-C coatings, and this ratio can be adjusted to control these properties. N. Boutroy et al. confirmed that amorphous carbon coatings that are introduced as a polymer substrate act as a gas barrier, inhibiting the penetration of oxygen and moisture [28,29], and M. Tamura et al. deposited a diamond-like carbon layer on stainless steel with a buffer layer and confirmed improved hydrogen barrier properties [30,31].

In this study, TiZrN coatings were doped with carbon by varying the laser output to investigate the hydrogen permeability according to the bonding environment and the surface roughness. XRD analysis and Rietveld refinement were used to confirm the crystal structure changes of carbon-doped TiZrN coatings, and the bonding state after carburization was confirmed through XPS. The microstructure of the coating surface was observed using TEM analysis, and surface roughness changes in the carbon-doped TiZrN coating were measured using AFM analysis. The hydrogen permeability of the carbon-doped TiZrN coating was also determined using a gas transmitter.

## 2. Materials and Methods

To enhance the adhesion of the coating layer on SUS304, the surface underwent a cleaning process, utilizing ultrasonication and ethanol to eliminate contamination, before the deposition. TiZrN coatings with a composition of Ti:Zr = 50:50 wt% were deposited onto the SUS304 substrate using RF/DC magnetron sputtering. The deposition was carried out under specific conditions: a base pressure of 1.0 × 10^−5^ Torr, a working pressure of 1.0 × 10^−2^ Torr, and a deposition temperature of 723 K for a duration of 6 h. For the cover of a carbon paste, a mixture of carbon paste, graphite powder (20 µm), and polyvinylidene fluoride (PVDF) at a ratio of 9:1 was created. The viscosity of the paste was adjusted using 1-methyl-2-pyrrolidinone as a solvent. This carbon paste was applied to the TiZrN coatings and dried at 80 °C. Subsequently, carburization of the coating surface was performed using a Nd:YAG pulsed laser ablation system (LSX-213), with the laser output being adjusted in 10% intervals from 20% to 50%. After the laser carburization process, any remaining carbon paste on the coating surface was removed through ultrasonication, acetone, and ethanol cleaning [32,33].

Phase analysis was conducted using an X-ray diffractometer (Rigaku, Ultima-IV, Woodlands, TX, USA) with a Cu target, scanning at a 0.02°/2θ step within the range of 20° to 80° at a rate of 1°/min. X-ray photoelectron spectroscopy (XPS) analysis (ESCALAB250, VG scientific, Waltham, MA, USA) was performed with an MXR1 gun-400 µm 15 kV spectrometer featuring an Al Kɑ source at the KBSi Busan Center. For microstructure analysis, coating specimens were prepared using a focused ion beam (FIB, Helios G4 UC, ThermoFisher Scientific, Waltham, MA, USA), and the cross-section of the coatings was observed using transmission electron microscopy (Cs-TEM, Titan Themis Z, ThermoFisher Scientific, USA). Atomic force microscopy (AFM) analysis was conducted, utilizing a Digital Instruments Multimode atomic force microscope controlled using a Nanoscope Ⅲ scanning probe microscope controller. Hydrogen permeation tests were carried out according to the differential pressure methods specified in ISO 15105-1 (determination of gas-transmission rate—differential pressure methods) [34].

## 3. Results and Discussion

**Figure 1:**
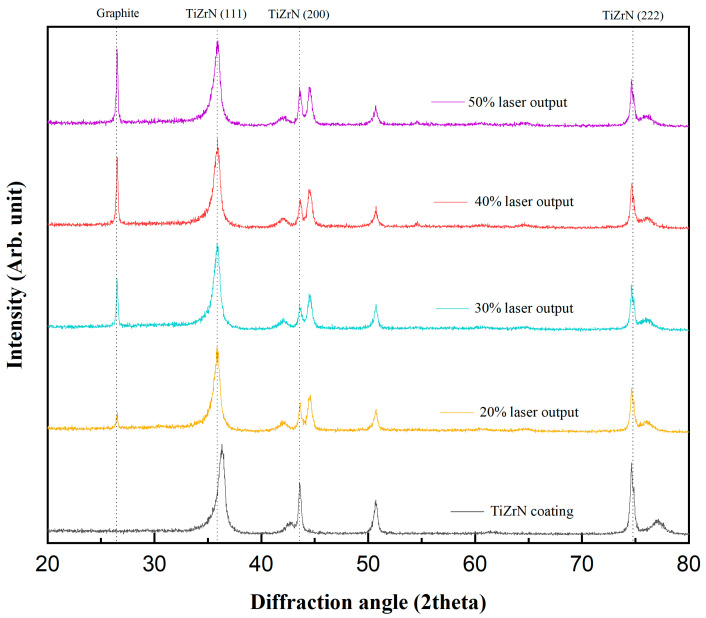
XRD patterns of carbon-doped TiZrN coatings with the laser output.

Figure 1 shows the XRD pattern of the TiZrN coating before and after carbon doping. The TiZrN coating has a face-centered cubic (FCC) crystal structure. The patterns showed peak diffractive angles at 36.46°, 42.36°, and 73.70° which correspond to the TiZrN (111), TiZrN (200), and TiZrN (222) planes, respectively. This represented the primary growth directions for TiZrN and confirmed the presence of strong (111) peak intensities, indicating the formation of columnar grains. The development of crystal structures was attributed to the addition energy of the deposition atoms on the substrate surface during film formation, which leads to an increased adatom mobility and, hence, a greater crystallinity of the films through a longer deposition time [35]. The diffraction peaks of the carbon-doped TiZrN coating were observed to shift to lower angles, indicating the substitution of carbon with nitrogen or the occupation of nitrogen vacancies by carbon due to the larger atomic radius of carbon. Rietveld refinement with the Sin^2^Ψ method was performed to evaluate the lattice distortion caused by carbon doping [33]. Before carburization, the lattice constant was 4.312 Å, but as expansion lattice distortion occurred due to the laser carburization, it showed 4.348 Å at 20% laser output, 4.366 Å at 30% laser output, 4.388 Å at 40% laser output, and 4.402 Å at 50% laser output. The grain size according to Scherrer’s equation is expressed as Table 1 follows [36]:

(1) $$D=\frac{K\lambda }{\beta \cos\theta }$$

Here, K is the Scherrer constant, λ is the wavelength of light used for the diffraction, *β* is the “full width at half maximum” of the sharp peaks, and *θ* is the angle measured. The Scherrer constant (K) in the above formula accounts for the shape of the particle and is generally taken to have a value of 0.9. The grain size, calculated through this equation, was 26.4 nm before carburization, but after carburization, it represented 25.7 nm at 20% laser output, 24.4 nm at 30% laser output, 20.1 nm at 40% laser output, and 18.3 nm at 50% laser output.

**Figure 2:**
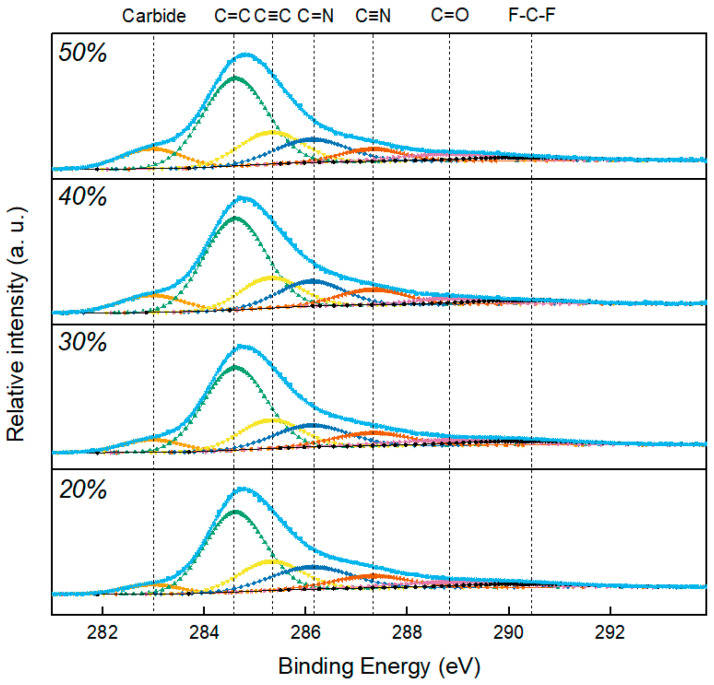
XPS C 1 s spectra of carbon-doped TiZrN coatings depending on the laser output.

Figure 2 describes the changes in the bonding behavior of the carbon-doped TiZrN coatings depending on the laser output, as observed in the XPS C 1 s diffraction. The lowest energy peak (283 eV) corresponds to the carbide peak (TiC, ZrC), and the peaks at 284.5 eV and 285.2 eV represent the sp^2^ and sp^3^ bonds of carbon, respectively. Carbon hybrid bonds were occupied at a high rate, and carbon nitride peaks were also observed. The peak at 290.4 eV is attributed to the CF bond from fluorine in the carbon paste. The formation of carbide increased with the laser output, indicating that the diffusion flow was more activated in the carbon paste as the amount of thermal energy increased [37,38]. The increase in the proportion of substitutional carbon with the rising thermal energy can be explained based on the atomic collision theory. It suggests that carbon atoms with high energy collide with (Ti, Zr)N molecules, leading to the conduction of thermal energy. Under conditions of low energy output (20% and 30% laser output), it is anticipated that the predominant phase formed is ZrC, given its lower formation energy. In contrast, at higher energy outputs (40% and 50% laser outputs), it is inferred that TiC, characterized by relatively higher formation energy, is likely to be the primary phase formed. The sp^2^/sp^3^ ratio was calculated to be 2.34 at 20% laser output in Table 2, while the 40% and 50% laser outputs showed ratios of 3.07 and 3.19, respectively; this is attributed to the accumulation of residual stress within the lattice due to the high thermal energy and carbon doping, leading to the formation of amorphous carbon over the solubility limit of carbon within the coating [39,40].

**Figure 3:**
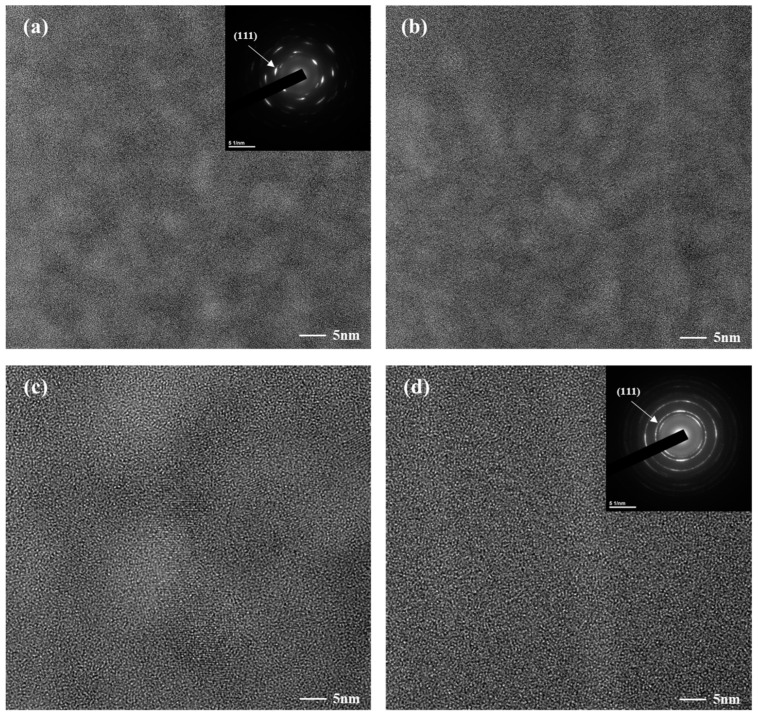
TEM images and SAED patterns of carbon-doped TiZrN coatings depending on the laser output: (**a**) 20% laser output, (**b**) 30% laser output, (**c**) 40% laser output, and (**d**) 50% laser output.

**Figure 4:**
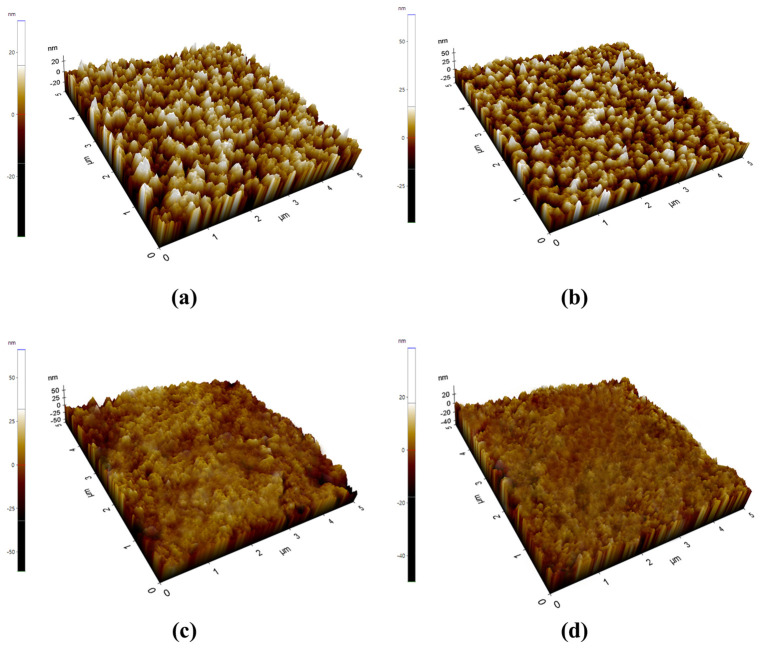
AFM morphologies for carbon-doped TiZrN coating: (**a**) 20% laser output, (**b**) 30% laser output, (**c**) 40% laser output, and (**d**) 50% laser output.

Figure 3 indicates the microstructural variation in the coatings using TEM images and SAED (selected area electron diffraction) patterns. The crystalline morphology observed in Figure 4a–d corresponds to the preferred directional growth, as evidenced by the X-ray diffraction (XRD) results. In all specimens, it was observed that the crystallite sizes were uniformly distributed without significant large voids. When the laser output was 20%, a clear dot pattern was observed in the SAED pattern, as shown in Figure 3a. However, as the laser output increased, a diffused ring pattern was observed at 50% laser output, indicating the formation of amorphous carbon in the intergranular structure [41,42]. In the TEM images, it was confirmed that the grain boundary became clearer as the laser output increased from 20% to 50%. Additionally, the location of amorphous carbon formation within the intergranular structure can be explained through the Harrison diffusion kinetics regimes. The Harrison Type-A kinetics regime (long diffusion time) refers to the situation where the average bulk diffusion length of tracer atoms coming from the tracer source is substantially greater than the grain size. The Harrison Type-B kinetics regime (intermediate time) refers to a situation where the average bulk diffusion length of the tracer atoms is less than the grain size. The Harrison Type-C kinetics regime (very short time) refers to a situation where the bulk diffusion length of the tracer atoms is less than the grain boundary width. As revealed in the XPS analysis, the local carbide phases (TiC and ZrC) exhibit lattice diffusion via short-circuit diffusion, suggesting diffusion via Harrison Type-B or Type-C mechanisms. Consequently, from the XPS and TEM results, it can be determined that the carbon atoms migrate via short-circuit diffusion through the grain boundaries, occupy the grain boundaries, and form an amorphous phase [43,44].

AFM was used to investigate the surface morphology of the carbon-doped TiZrN coating with the laser output, as shown in Figure 4. The roughness average (Ra) is the arithmetic average of the absolute values of the profile height deviations from the mean line, recorded within the evaluation length. Simply put, the Ra is the average of a set of individual measurements of a surface’s peaks and valleys. The Ra value was 12.913 nm at 20% laser output and decreased with the increasing laser output to 11.214 nm at 30%, 8.462 nm at 40%, and 7.127 nm at 50%. As the laser power increased, the amount of carbon doping increased and the grain size of the (Ti, Zr)(C, N) decreased, and amorphous carbon was formed in the TiZrN coatings. Consequently, from these results, we determined that amorphous carbon-embedded (Ti, Zr)(C, N) was formed in the TiZrN coating.

The hydrogen permeability of the carbon-doped TiZrN coating was measured according to the ISO 15105-1 standard. The gas permeability is expressed as follows [43,45,46]:

(2) $$\phi =J\cdot \frac{d}{A}\cdot \Delta p^{n}$$

Here, *J* represents the hydrogen permeation for a specimen with surface area *A* (6.6 × 10^−4^ m^2^) and thickness d (2.5 × 10^−6^ m for the coating and 1.0 × 10^−4^ m for the substrate) under a pressure differential, ΔP. The parameter n indicates the mode of permeation, with n = 0.5 for diffusion-limited and n = 1.0 for surface-limited permeation. The hydrogen permeation of a metal-coated specimen is diffusion-limited in the pressure range of 104 Pa–105 Pa, and therefore, the overall permeation, *J*, can be expressed using the following equation:

(3) $$J=\phi \cdot \left[{\left(P_{H_{2},i}\right)}^{0.5}-{\left(P_{H_{2},o}\right)}^{0.5}\right]\cdot A/d$$

Here, Ø represents the permeation coefficient, (4.0 × 10^5^ Pa) is the hydrogen pressure at the inlet, and (1.0 × 10^−6^ Pa) is the hydrogen pressure at the outlet. The measured hydrogen permeability can be expressed as an Arrhenius function of temperature, as shown in Equation (4), which allows for the calculation of the activation energy (*Eϕ*) corresponding to the slope and the preexponential factor (*ϕ*_0_) corresponding to the intercept.

(4) $$\phi =\phi_{0}\exp\left[-\frac{E_{\phi }}{RT}\right]$$

**Figure 5:**
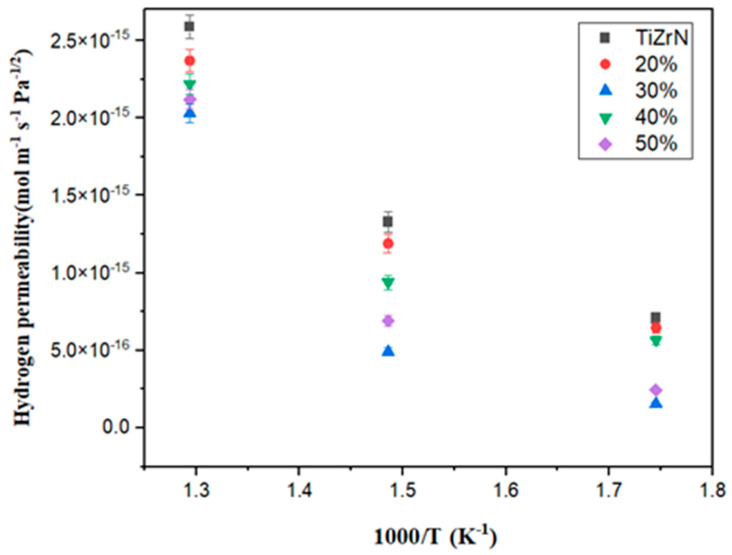
Hydrogen permeability of carbon-doped TiZrN coatings depending on the laser output.

The hydrogen permeability as a function of temperature for the TiZrN coating and carbon-doped TiZrN coating was calculated using Equations (5) and (6), respectively, and is presented in Figure 5.

(5) $$\phi_{TiZrN}=\left(9.48\times 10^{-14}\;\mathrm{mol}\cdot m^{-1}\cdot s^{-1}\cdot {\mathrm{Pa}}^{-1/2}\right)\times \exp\left[-\frac{23.46\;\mathrm{kJ}\cdot {\mathrm{mol}}^{-1}}{RT}\right]$$

(6) $$\phi_{C-TiZrN}=\left(2.55\times 10^{-12}\;\mathrm{mol}\cdot m^{-1}\cdot s^{-1}\cdot {\mathrm{Pa}}^{-1/2}\right)\times \exp\left[-\frac{46.65\;\mathrm{kJ}\cdot {\mathrm{mol}}^{-1}}{RT}\right]$$

As shown in Figure 5, the hydrogen permeability in all temperature ranges (573 K, 673 K, 773 K) decreased after carburization compared to before carburization. At high laser output (40%, 50%), which is considered to have formed amorphous carbon-embedded nanocomposites, the hydrogen permeability decreased by up to 78% (573 K), 63% (673 K), and 21% (773 K) compared to before carburization. This is judged to be due to the formation of an intergranular structure through carbon doping in the TiZrN coatings, which resulted in the presence of amorphous carbon in the grain boundary, and the surface roughness decreased as it gradually changed to a carbon-embedded nanocomposite.

## 4. Conclusions

In this study, carbon was doped to reduce the hydrogen permeability of the TiZrN coating, and the formation of amorphous carbon-embedded (Ti, Zr)(C, N), surface roughness, and hydrogen permeability in the carbon-doped TiZrN coating were investigated. Using Scherrer’s equation, we calculated a decrease in grain size in the TiZrN coating, from 26.49 nm before carbon doping to 18.31 nm after carbon doping, demonstrating nanocrystalline refinement and a transition to an intergranular structure. At low energy outputs (20% and 30% laser output), it is anticipated that the predominant phase formed is ZrC, given its lower formation energy. In contrast, at high energy outputs (40% and 50% laser output), it is inferred that TiC, characterized by relatively a higher formation energy, is likely to be the primary phase formed. The sp^2^/sp^3^ ratio was 1.23 at 20% output and 1.31 at 30% output, while it was 2.64 at 40% and 2.89 at 50%, meaning that amorphous carbon was formed. For the TEM and SAED patterns, the surface microstructure of the carbon-doped TiZrN coatings transitioned to an intergranular structure, indicating the creation of amorphous carbon-embedded (Ti, Zr)(C, N) in the coating. The surface roughness (Ra) decreased from 14.13 nm before carburization to 8.462 nm (40% laser power) and 7.12 nm (50% laser power) after forming the amorphous carbon-embedded nanocomposite. Furthermore, the hydrogen permeability was decreased in all temperature ranges from 573 K to 773 K, and was especially reduced by 78% at 50% laser power. This is attributed to the amorphous carbon-embedded nanocomposite interrupting the hydrogen path and increasing the hydrogen adsorption activation energy through the lubricating effect of the coating surface.

## Figures and Tables

**Table 1:** Lattice constant and grain size of carbon-doped TiZrN coatings.

Laser Output	Lattice Constant	Grain Size
0% (TiZrN)	4.312 Å	26.49 nm
20%	4.348 Å	25.78 nm
30%	4.366 Å	24.45 nm
40%	4.388 Å	20.14 nm
50%	4.402 Å	18.31 nm

**Table 2:** Carbide and sp^2^/sp^3^ ratio of carbon-doped TiZrN coatings depending on the laser output.

Laser Output	Carbide	Sp^2^/Sp^3^ Ratio
20%	4%	1.23
30%	6.2%	1.31
40%	6.9%	2.64
50%	7.5%	2.89
